# Effects of Afternoon Nap Deprivation on Adult Habitual Nappers' Inhibition Functions

**DOI:** 10.1155/2018/5702646

**Published:** 2018-03-22

**Authors:** Qingwei Chen, Taotao Ru, Minqi Yang, Pei Yan, Jinghua Li, Ying Yao, Xiaoran Li, Guofu Zhou

**Affiliations:** ^1^Research Center of Light and Physio-Psychological Health, School of Psychology, South China Normal University, Guangzhou 510631, China; ^2^National Center for International Research on Green Optoelectronics, South China Normal University, Guangzhou 510006, China; ^3^Shenzhen Guohua Optoelectronics Tech. Co. Ltd., Shenzhen 518110, China; ^4^Guangdong Provincial Key Laboratory of Optical Information Materials and Technology & Institute of Electronic Paper Displays, South China Academy of Advanced Optoelectronics, South China Normal University, Guangzhou 510006, China

## Abstract

Multiple studies have established the effects of afternoon naps on cognition. However, relatively few studies have investigated the domain of executive functions. Moreover, the effects of napping on inhibition are far from conclusive. The present study employed adult habitual nappers to investigate the effects of afternoon nap deprivation on response-based inhibition assessed by a Go/No-go task and stimulus-based inhibition assessed by a Flanker task and on alertness assessed by a psychomotor vigilance test (PVT) and the Karolinska Sleepiness Scale (KSS). The results showed that afternoon nap deprivation significantly decreased participants' accuracy and reaction speed for the Go/No-go task but not for the Flanker task. In addition, participants' alertness was significantly impaired after nap deprivation in terms of increased subjective sleepiness and worse PVT performance. Task-specific effects of napping on inhibition were demonstrated. The implications of the results are discussed.

## 1. Introduction

Daytime sleepiness is a universal issue in our everyday life. Particularly in the early afternoon, many people struggle to maintain the required alertness level. Napping, as a practical countermeasure to daytime sleepiness induced by insufficient sleep as well as homeostatic sleep drive, has also been reported to significantly influence human cognitive performance, emotion regulation, decision making, social interactions, and traffic driving [1–3]. Of these areas, multiple studies have investigated and established the effects of a nap on cognition during biological night and day (see reviews [1–4]). As suggested by Ficca et al. [2], laboratory studies about the effects of napping on cognition should pay more attention to high-order cognitive functions, especially executive functions. Recently, substantial effort has been undertaken to fill this gap.

Research about the high-order cognitive effects of a short daytime nap has mainly focused on memory and learning [5–7], though relatively fewer studies have investigated the effects of nap on executive functions (i.e., inhibition, working memory, and cognitive flexibility) [1, 8]. The available evidence has consistently demonstrated that a short daytime nap has beneficial effects on working memory [9–11] and cognitive flexibility [12, 13]. Regarding the napping effect on inhibition, however, mixed results have emerged. Some studies have revealed beneficial effects of napping on inhibition. For example, a short daytime nap increased the accuracy of Flanker task for preschool children [14] and a nocturnal nap improved Go/No-go task performance for adults [15]. However, there are also studies suggesting no effect of napping on inhibition. For instance, response inhibition evaluated by Go/No-go task was not affected by sleep restriction by means of nap deprivation and delayed bedtime in early childhood [16]. Several studies have even reported the deleterious influence of napping on inhibition. Lam et al. [17] found that a nap-deprivation group showed significant improvements in Go/No-go task compared with a napping-as-usual group of preschoolers.

These inconsistencies may be due to several factors. First, various types of tasks employed in different studies could explain these contradictory findings. Some studies employed a Flanker task [14] to assess inhibition, while some utilized Go/No-go tasks [15–17]. It is difficult to directly compare the findings between these studies, since different tasks and study paradigms were employed. Thus, the first purpose of the current study was to investigate the effects of daytime nap on inhibition by using both Flanker and Go/No-go tasks within one study.

In addition, the benefits of a daytime nap may also be influenced by the manipulation of napping itself, in particular with regard to the nap duration and napping habits [1, 2, 4]. It is well established that a short afternoon nap is more effective than a long one to counteract the postlunch dip in alertness and performance because a long nap might cause sleep inertia [18, 19]. Lam et al. [17] also suggested that worse inhibition performance may be caused by sleep inertia immediately after a nap. Moreover, napping habits would be another factor influencing the effect of nap on mental performance. Previous study suggests that habitual nappers benefit more from a nap than individuals who are not used to taking a midday nap [20]. Recent evidence has confirmed that napping habits could mediate the relationship between naps and cognition [21, 22]. As the case for China, most people prefer to take a short nap at noon or in the early afternoon as a practical option to alleviate the postlunch dip or as compensation for insufficient sleep at night. Thus, it is of value to perform a daytime nap-deprivation study in the adult population in China. Combined with the fact that research about the effects of daytime nap deprivation on cognition in adult habitual nappers is scarce, the second purpose of the current study is to clarify the influence of regular nap deprivation on habitual nappers' inhibition.

As a specific kind of sleep restriction, the line of research about sleep deprivation and sleep restriction would provide us with some encouraging evidence about nap deprivation, especially daytime nap deprivation. In a meta-analysis, Lim and Dinges [23] concluded that sleep restriction causes little to no impairment on response inhibition tasks, such as Go/No-go task. However, recent evidence seems to not confirm this expectation. Both one night of total sleep deprivation [24–27] and four days of partial sleep restriction [28] have been shown to worsen performance on Go/No-go task. Additionally, another line of research employing Flanker task found similar results [27, 29, 30]. All these studies were conducted at night. However, whether this conclusion could be generalized to daytime nap deprivation requires empirical investigation.

In the current study, we investigated the effects of afternoon nap deprivation on inhibition assessed by Go/No-go task and Flanker task in habitual adult nappers. In addition, the influence of nap deprivation on participants' subsequent objective and subjective alertness was also investigated using a psychomotor vigilance test (PVT) [31] and the Karolinska Sleepiness Scale (KSS) [32], respectively. Based on findings in previous studies, it was suggested that sleepiness induced by sleep deprivation has domain-specific influences on cognitive performance [33–36]. In a recent meta-analysis, Wickens et al. [37] have demonstrated that sleep disruption led to less deterioration of complex cognitive task performance compared to simple cognitive task (such as PVT) performance. Hence, we predicted that nap deprivation would decrease subjective alertness and worsen the performance of the PVT, Go/No-go task, and Flanker task. Moreover, inhibition performance in Go/No-go task and Flanker task would be less impaired than simple attentional vigilance performance in the PVT task by nap deprivation.

## 2. Method

### 2.1. Participants

Twenty healthy adults (8 males; 18–24 years old) participated in the study. All participants gave written informed consent before formal study. The study was performed in agreement with the regulations of the Ethics Committee on Research involving Humans at the South China Normal University.

### 2.2. Screening Procedure and Protocol

Healthy volunteers were recruited via advertisements at the local university. Only participants who habitually had a nap of 30–40 minutes at afternoon (13:00–14:00 h) were included in this study. All potential participants were requested to complete online questionnaires before the formal study, including questions about demographics, napping behavior, physical and mental health, nighttime sleep quality, and chronotype.

Physical and mental health problems were screened according to the participants' self-report ratings on the General Medical Questionnaire [38]. No participants reported (1) shift-work or travel to a different time zone in the last 3 months, (2) drug consumption or smoking, (3) body mass index >25 or <20, (4) <7 hours or >9 hours spent in bed at night, (5) extremely late or extremely early chronotype on the Munich Chronotype Questionnaire (MCTQ) [39], (6) score of the Beck Depression Inventory >8 [40], and (7) score of Pittsburgh Sleep Quality Index >5 [41].

Two days before the lab study day, each participant arrived in the laboratory and practiced performing all the experimental tasks. Participants were asked to follow their regular night sleep schedule and to refrain from consuming caffeine or alcohol on each experimental day as well as on the day prior to an experimental day. We used wrist activity monitors (Actiwatch, Philips Respironics) to record participants' sleep duration during the night and daily activities before experiment days.

### 2.3. Design and Materials

A one-factor nap intervention (nap versus nap-deprivation) within-subject design was adopted in the current study. All participants were tested in the laboratory room on three separate and nonconsecutive days. The first of these was an adaptation day to make participants familiar with the laboratory environment and get used to napping in it. On the other two days, two experimental manipulations with either nap condition or nap-deprivation condition were performed with an interval of at least 3 days. The order of nap manipulations was counterbalanced across participants.

**Figure 1:**
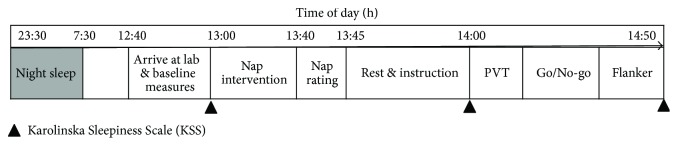
Schematic representation of one experimental session; the exact times of night sleep and daytime nap were identified according to each participant's schedule. The three cognitive tasks were performed in pseudorandom order on one specific experimental day and were identical for participants across nap and nap-deprivation conditions. Among these tasks, PVT always came first, and the sequence of the Go/No-go and Flanker tasks was counterbalanced across the participants.

Figure 1 represents the procedure of one experimental session (one instance for a participant who fell asleep at 23:30 h, woke up at 7:30 h, and had a 40 min (time in bed) intervention at 13:00 h). All participants were instructed to arrive at the laboratory before 12:40 h, baseline measurement about subjective alertness was performed with short questionnaires (see below) at the beginning of each experimental session, and then participants were assigned to receive either the nap or nap-deprivation condition. In the nap condition, participants received a 40 min opportunity to nap on a bed in the lab room in dim light (near darkness) while being monitored by an assistant in another room via a webcam. In the nap-deprivation condition, participants were required to sit in the lab room and stay awake for 40 minutes. In this condition, participants were free to read paper books and walk around in the lab room during this period. An assistant stayed with participants to remind them not to doze. After the 40 min intervention (either nap or nap deprivation), all participants received a 20 min opportunity for free activities in the lab room, to minimize any potential effects of sleep inertia.

The test session started at 14:00 h with 2 min for questionnaires about participants' current alertness. Three computerized cognitive tasks were performed after this. Among these tasks, PVT always came first, and the sequence of the Go/No-go and Flanker tasks was counterbalanced across the participants. All test blocks started with instructions and a few practice trials. Reaction time (RT) and accuracy were both emphasized in the instructions. Subjective alertness was measured again after the final task.

### 2.4. Assessment of Subjective Alertness and Nap Duration

Subjective alertness was assessed with the KSS [32] at the beginning (pretest) and end (posttest 1) of the nap intervention, as well as at the end (posttest 2) of the experimental session. Additionally, participants in the nap condition were requested to rate their nap quality (i.e., how long did it take them to fall asleep and awaken fully).

### 2.5. Cognitive Tasks

There were three computerized tasks in total in the present study. The auditory version of psychomotor vigilance test (PVT) was adopted to evaluate objective alertness [31]. The Go/No-go task was used to assess response inhibition capacities [25]. In addition, the Flanker task was utilized to evaluate conflict monitoring and resolution [42].

#### 2.5.1. Auditory PVT

During a 10 min auditory PVT, participants were required to press the SPACE key as soon as possible when they heard a beep (100 ms, 1000 Hz) through headphones. Beeps were presented with a randomized interstimulus interval (ISI) ranging from 1000 ms to 9000 ms.

#### 2.5.2. Go/No-Go Task

Stimuli included a capital letter “M” or “W” which appeared in the center of a black screen. Participants were instructed to press the SPACE key as soon as possible for the target “W” (go condition) but not for the nontarget “M” (no-go condition). A fixation cross was presented for 500 ms, followed by a 1000 ms stimulus and with a randomized ISI ranging from 1000 ms to 1500 ms. Each block included 200 trials.

#### 2.5.3. Flanker Task

The target stimulus was an arrow pointing either to the left or to the right at the center of the black screen, surrounded by other arrows. The surrounding Flanker arrows pointed in the same direction as the target arrow in the congruent condition, while the surrounding Flanker arrows pointed in the opposite direction as the target arrow in the incongruent condition. Participants were told to indicate the direction of the target arrow by pressing “J” or “F” on the keyboard, while neglecting the direction of the Flanker arrows. Each block included 80 congruent and 80 incongruent trials.

### 2.6. Data Analysis

The data from two participants (1 female and 1 male) were missing due to errors when saving data. Thus, eighteen valid datasets (7 male) were used for final statistical analysis. RTs in inaccurate trials and outliers (more than three standard deviations from the mean) were removed before subsequent analysis for Go/No-go and Flanker tasks. The data from one participant (female) was discarded because of outliers (outside three standard deviations from mean) on RT on the Flanker task, which led to seventeen valid datasets (7 males, mean age 21.35 ± 1.87 years). For the PVT data, the overall reaction time, the 10% fastest RTs, and 10% slowest RTs were computed separately after discarding RTs shorter than 100 ms (false start) and trials without a response. Lapses were calculated as the total number of trials without a response and trials with RTs longer than 500 ms. For the Flanker task, average reaction time and accuracy in both congruent and incongruent trials were calculated separately.

For all analyses, SPSS 16.0 was used. Since subjective sleepiness was assessed multiple times. Testing time (pretest, posttest 1, and posttest 2) was added as a within-subjects factor in the analysis on these variables. For the Flanker task, a two-way repeated measure ANOVA with nap intervention (nap versus nap-deprivation) and congruency (congruent versus incongruent) as within-subject factors was conducted on average speed and accuracy. For other task performances, the data were analyzed by a one-way repeated measure ANOVA with the within-subject factors nap intervention (nap versus nap-deprivation) for both reaction speed and accuracy (lapse in the PVT task).

## 3. Results

### 3.1. Sleep Duration

The average nap duration for the participants in the nap condition was 31.76 ± 6.60 (*M* ± SD) minutes and they reported that it took 7.82 ± 2.72 minutes to fully wake up. Analysis of the sleep duration of the previous night indicated that there were no significant differences between nap (440.67 ± 39.84 min), nap-deprivation (448.11 ± 37.45 min), and adaptation conditions (447.33 ± 35.89 min), *ps* > 0.05.

### 3.2. Subjective Alertness

Repeated-measures ANOVA on the KSS scores revealed a significant effect of nap intervention [*F*(1, 16) = 71.82, *p* < 0.001, *η*^2^ = 0.82], test time [*F*(2, 32) = 6.94, *p* = 0.006, *η*^2^ = 0.30], and the nap intervention × test time interaction [*F*(2,32) = 17.12, *p* < 0.001, *η*^2^ = 0.52]. Post hoc tests indicated that participants' subjective alertness at baseline did not significantly differ between nap or nap-deprivation conditions [4.76 ± 0.66 versus 4.88 ± 0.70, *p* > 0.05], while alertness level was, on average, lower at either posttest 1 [3.88 ± 1.32 versus 6.71 ± 1.31] or posttest 2 [2.29 ± 0.92 versus 5.53 ± 2.45] in the nap-deprivation condition compared to the nap condition (*ps* < 0.05).

### 3.3. Task Performance

#### 3.3.1. Psychomotor Vigilance Task

**Figure 2:**
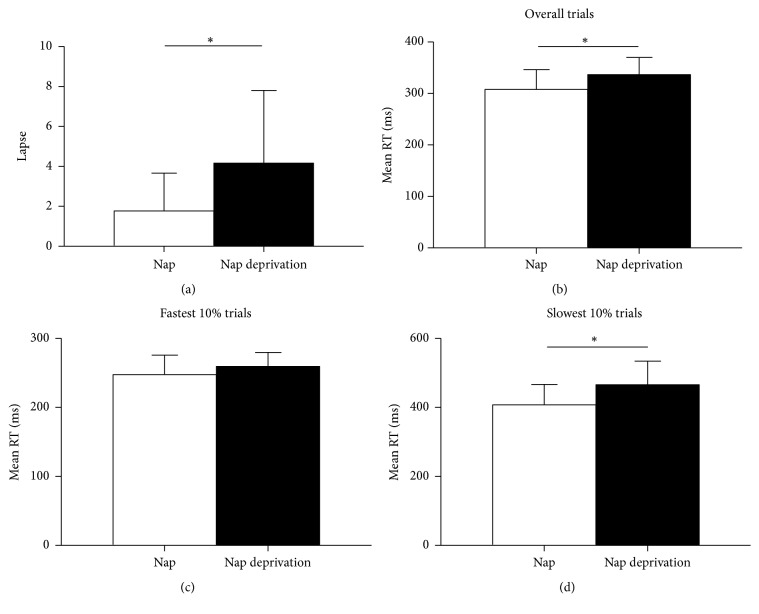
PVT task performance. (a) Lapse; (b) average reaction time for all valid trials; (c) average reaction time for the fastest 10% of the trials; and (d) average reaction time for the slowest 10% of the trials for the nap condition (white bars) and the nap-deprivation condition (black bars). Error bars indicate ±1 standard deviation of the mean. ^*∗*^*p* < 0.05.

Figure 2 shows the average performance on the PVT task in the nap and nap-deprivation conditions. Lapses (Figure 2(a)) were significantly higher in the nap-deprivation condition than the nap condition, *F*(1,16) = 5.13, *p* = 0.04, *η*^2^ = 0.24. Likewise, the average reaction time for overall PVT trials (Figure 2(b)) as well as the slowest 10% of the trials (Figure 2(d)) significantly decreased after nap deprivation, *F*(1, 16) = 5.21, *p* = 0.04, *η*^2^ = 0.25 and *F*(1,16) = 6.87, *p* = 0.02, *η*^2^ = 0.30, respectively. However, the main effect of nap intervention did not reach significance for the average speed of the fastest 10% of the trials (Figure 2(c)), *F*(1,16) = 2.22, *p* = 0.16, *η*^2^ = 0.12.

#### 3.3.2. Go/No-Go Task

**Figure 3:**
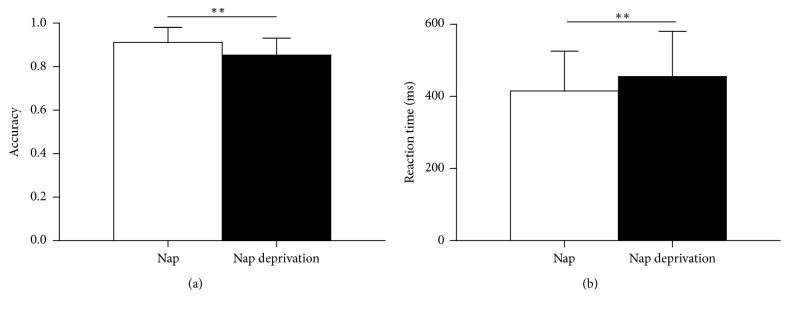
Go/No-go task performance. (a) Accuracy for the nap condition (white bars) and the nap-deprivation condition (black bars). (b) Average reaction time. Error bars indicate ±1 standard deviation of the mean. ^*∗∗*^*p* < 0.01.

The performance on the Go/No-go task is shown in Figure 3. Participants in the nap-deprivation condition made significantly more errors on the Go/No-go task than in the nap condition (0.85 ± 0.08 versus 0.91 ± 0.07), *F*(1,16) = 8.95, *p* = 0.009, *η*^2^ = 0.36. Moreover, the effect of nap versus nap-deprivation on reaction speed reached significance as well, *F*(1,16) = 8.82, *p* = 0.009, *η*^2^ = 0.36. The participants responded faster in the nap condition (415.28 ± 110.15 ms) than in the nap-deprivation condition (455.16 ± 125.76 ms).

#### 3.3.3. Flanker Task

**Figure 4:**
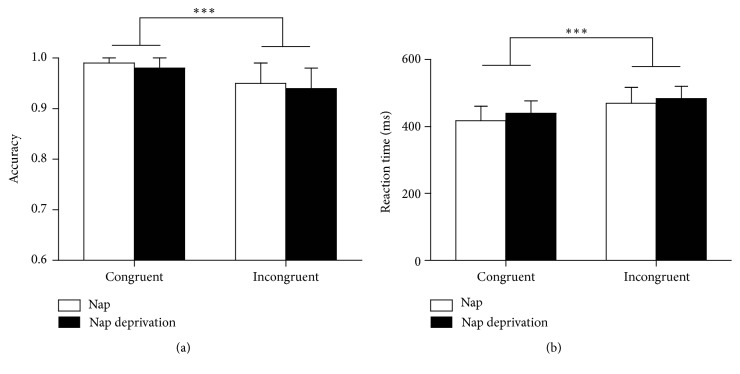
Flanker task performance. (a) Accuracy for the nap condition (white bars) and the nap-deprivation condition (black bars) for congruent and incongruent trials. (b) Average reaction time. Error bars indicate ±1 standard deviation of the mean. ^*∗∗∗*^*p* < 0.001.

Figure 4 shows the results on the Flanker task. A two-way repeated-measures ANOVA with nap intervention (nap versus nap-deprivation) and congruency (congruent versus incongruent) as within-subject factors for accuracy (see Figure 4(a)) revealed a significant main effect for congruency, *F*(1,16) = 26.27, *p* < 0.001, *η*^2^ = 0.62. Participants made significantly more accurate responses on congruent trials (0.99 ± 0.02) than on incongruent ones (0.95 ± 0.05). The nap intervention did not have a significant effect on accuracy [*F*(1,16) = 3.68, *p* = 0.07, *η*^2^ = 0.19], nor did the interaction between nap intervention and congruency [*F*(1,16) = 0.26, *p* = 0.62, *η*^2^ = 0.02].

Similarly, the two-way ANOVA of reaction speed (see Figure 4(b)) revealed a marginally significant difference for congruency [*F*(1,16) = 206.08, *p* < 0.001, *η*^2^ = 0.93], with participants responding faster in the congruent condition (428.61 ± 41.40 ms) than in the incongruent condition (476.94 ± 42.12 ms). No other significant effects were revealed (*ps* > 0.05).

## 4. Discussion

By enrolling adult habitual nappers, the current study investigated the effect of nap deprivation on alertness and inhibition functions. The results suggest that a short nap deprivation significantly increased participants' subjective sleepiness and worsened the vigilance performance assessed with the PVT task, while only significantly impaired participants' executive inhibition assessed with Go/No-go task not the Flanker task. Our findings transfer the conclusions drawn from nocturnal sleep restrictions on inhibition to short daytime nap deprivation. Moreover, unlike previous studies about daytime nap deprivation that mainly focused on preschool children [14, 16, 17], this study put an emphasis on healthy adults with a long-term habit of afternoon naps, which is quite different from those from societies without nap habits. Last but not least, extending previous studies, we employed Go/No-go task and Flanker task simultaneously in one study to explore the potential task-specific effect of daytime nap deprivation on inhibition functions for habitual adult nappers.

Multiple studies have investigated the effects of daytime naps on higher executive functions. The results of these studies, however, are rather inconsistent [13, 43–45]. The current findings reveal that a short afternoon nap deprivation differentially influenced participants' executive function of response inhibition when assessed with a Go/No-go task and the function of conflict monitoring when assessed with a Flanker task. For the Go/No-go task, participants' accuracy and reaction speed were both significantly decreased after a short afternoon nap deprivation. This finding is partially in line with those from previous studies that reported either positive, null, or negative effects of a short nap on response inhibition [15–17]. Although several nocturnal studies have reported significant impairments of sleep deprivation on performance monitoring [14, 27, 29, 30], the current results revealed that neither accuracy nor reaction speed in the Flanker task was affected by a short daytime nap deprivation.

It is possible that differences in individual characteristics and study paradigms could partly explain these contradictory findings between previous studies and those from the current one. First, participants employed in most of the previous studies were preschool children [14, 16, 17], while adult habitual nappers were employed in the current study. Moreover, participants in the study by Schumacher et al. [16] were sleep-restricted during the night before the experiment day. In addition, while night sleep deprivation was reported to significantly deteriorate Flanker performance [27, 29, 30], this finding, however, did not transfer to the daytime situation, suggesting participants' mental functions would be differentially influenced by night sleep deprivation and daytime nap deprivation. Several studies have suggested that frontal brain regions and attentional networks are disrupted during sleep deprivation (see reviews [23, 35]). Considering that executive inhibition and monitoring are modulated by the anterior cingulate cortex (ACC) region of the prefrontal cortex (PFC) [8], one could speculate that a short daytime nap deprivation would selectively moderate brain activities involved in prefrontal cortex- (PFC-) oriented tests.

In addition to executive inhibition, the current study also investigated the effect of nap deprivation on participants' alertness level. The results showed that a short afternoon nap deprivation significantly elevated subjective sleepiness evaluated by KSS for adult habitual nappers. Participants felt sleepier after nap deprivation and reported significantly higher sleepiness throughout the subsequent test sessions than those who had taken a regular afternoon nap. The current findings are comparable with those from previous studies suggesting a short daytime nap significantly increased participants' alertness in terms of decreased sleepiness and lower alpha and theta power [44, 46–48].

Moreover, the current results showed that a short afternoon nap deprivation significantly deteriorated participants PVT performance, which was used to measure objective alertness level. More response lapses and lower reaction speeds in the PVT task were shown for participants who did not take a regular afternoon nap. These findings confirmed the expectation that performance on a simple reaction time task would benefit more from a daytime nap than relatively complicated executive functions [22, 45]. However, Slama et al. [13] did not report significant benefits of an afternoon nap on PVT performance. Again, the differences in individual characteristics and study paradigms make it difficult to directly compare the findings of the Slama et al. [13] study and the current one. Specifically, participants without nap habit were used in Slama et al. [13] study and they were tested after one hour and half past two hours after nap manipulation. These findings may suggest that the relationship between the napping time and the test time could mediate the alerting effect of a short afternoon nap.

Regarding the effect of a short afternoon nap deprivation on different cognitive domains, the current findings revealed that no consistent pattern emerged with respect to the effects of nap deprivation on simple vigilance and higher executive inhibition functions. A short afternoon nap deprivation significantly decreased participants PVT and Go/No-go performance, but not the performance on the Flanker task. There are also some studies that reported differential influences of a short afternoon nap on different cognitive tasks within one study paradigm [13, 43, 45]. Together, these findings suggest that the effect of a short afternoon nap on cognitive performance depends on the type of task. In addition to cognitive domains, task difficulty would be another potential factor mediating the effect of a short afternoon nap on cognition. Although the Go/No-go task and the Flanker task were both used to measure participants' executive inhibition ability, it is notable that the difficulties of the tasks themselves were different. Participants performed with relatively high accuracy (higher than 90%) and/or response speed for the Flanker task but not for the Go/No-go task in both nap and nap-deprived conditions. Moreover, Zhang and colleges [45] did not reveal the positive effects of a daytime nap on executive function measured with either a short-term memory recall task or an arithmetic calculation task. However, two recent studies have reported benefits of napping on task-switching tasks used to assess executive function [12, 13]. Most of the current studies have restricted their tasks to a limited range of task difficulty levels. Hence, this may lead to an open question on whether the effect of a short nap or sleep on cognitive functions would be mediated simultaneously by the type of task and the task difficulty, which was preliminarily answered by one of our follow-up studies [49]. In this follow-up study, we mainly focus on the beneficial effects of habitual daytime nap on alertness, mood, and cognitive performance, while we put more emphasis on the undesirable influences of habitual daytime nap deprivation on alertness and inhibition functions in the current investigation.

There are also some limitations in the current study. First, we did not employ objective measures (i.e., EEG) of napping behavior in the nap condition; therefore, the parameters of napping (i.e., duration and structure) remain unknown in the present study. Recently, Lau et al. [9] found that rapid-eye-movement sleep was associated with the enhancement of working memory performance after a daytime nap. Future studies could measure electrophysiological activities during the napping phase with polysomnography (PSG) to test the relationship between nap architecture and subsequent inhibition performance. Secondly, the participants were not totally blinded to the manipulation of napping, which may potentially bias the results, but only the subjective ratings of alertness. Thirdly, all the participants in the current study were adults with a long-term habit of afternoon naps. It remains unknown whether the current findings could be extended to nonhabitual nappers. Currently, it would be better for people to take a regular nap before they carry out work that demands alertness and inhibition, especially for those who have an afternoon nap habit.

## References

[1] Faraut B., Andrillon T., Vecchierini M.-F., Leger D. (2017). Napping: A public health issue. From epidemiological to laboratory studies. *Sleep Medicine Reviews*.

[2] Ficca G., Axelsson J., Mollicone D. J., Muto V., Vitiello M. V. (2010). Naps, cognition and performance. *Sleep Medicine Reviews*.

[3] Mantua J., Spencer R. M. C. (2017). Exploring the nap paradox: are mid-day sleep bouts a friend or foe?. *Sleep Medicine*.

[4] Hilditch C. J., Dorrian J., Banks S. (2017). A review of short naps and sleep inertia: do naps of 30 min or less really avoid sleep inertia and slow-wave sleep?. *Sleep Medicine*.

[5] Konrad C., Seehagen S., Schneider S., Herbert J. S. (2016). Naps promote flexible memory retrieval in 12-month-old infants. *Developmental Psychobiology*.

[6] Lemos N., Weissheimer J., Ribeiro S. (2014). Naps in school can enhance the duration of declarative memories learned by adolescents. *Frontiers in Systems Neuroscience*.

[7] Urbain C., De Tiège X., Op De Beeck M. (2016). Sleep in children triggers rapid reorganization of memory-related brain processes. *NeuroImage*.

[8] Diamond A. (2013). Executive functions. *Annual Review of Psychology*.

[9] Lau E. Y. Y., Wong M. L., Lau K. N. T., Hui F. W. Y., Tseng C.-H. (2015). Rapid-eye-movement-sleep (REM) associated enhancement of working memory performance after a daytime nap. *PLoS ONE*.

[10] Lo J. C., Lee S. M., Teo L. M., Lim J., Gooley J. J., Chee M. W. L. (2017). Neurobehavioral impact of successive cycles of sleep restriction with and without naps in adolescents. *SLEEP*.

[11] Waterhouse J., Atkinson G., Edwards B., Reilly T. (2007). The role of a short post-lunch nap in improving cognitive, motor, and sprint performance in participants with partial sleep deprivation. *Journal of Sports Sciences*.

[12] Kaida K., Takeda Y., Tsuzuki K. (2013). The effects of short afternoon nap and bright light on task switching performance and error-related negativity. *Sleep and Biological Rhythms*.

[13] Slama H., Deliens G., Schmitz R., Peigneux P., Leproult R. (2015). Afternoon nap and bright light exposure improve cognitive flexibility post lunch. *PLoS ONE*.

[14] Cremone A., McDermott J. M., Spencer R. M. C. (2017). Naps enhance executive attention in preschool-aged children. *Journal of Pediatric Psychology*.

[15] Tempesta D., Cipolli C., Desideri G., De Gennaro L., Ferrara M. (2013). Can taking a nap during a night shift counteract the impairment of executive skills in residents?. *Medical Education*.

[16] Schumacher A. M., Miller A. L., Watamura S. E., Kurth S., Lassonde J. M., LeBourgeois M. K. (2017). Sleep Moderates the Association Between Response Inhibition and Self-Regulation in Early Childhood. *Journal of Clinical Child & Adolescent Psychology*.

[17] Lam J., Koriakin T., Scharf S. M., Mason T. B. A., Mahone E. M. (2015). Does increased consolidated nighttime sleep facilitate attentional control? A pilot study of nap restriction in preschoolers. *Journal of Attention Disorders*.

[18] Takahashi M., Fukuda H., Arito H. (1998). Brief naps during post-lunch rest: Effects on alertness, performance, and autonomic balance. *European Journal of Applied Physiology*.

[19] Tietzel A. J., Lack L. C. (2002). The recuperative value of brief and ultra-brief naps on alertness and cognitive performance. *Journal of Sleep Research*.

[20] Evans F. J., Cook M. R., Cohen H. D., Orne E. C., Orne M. T. (1977). Appetitive and replacement naps: EEG and behavior. *Science*.

[21] Kurdziel L., Duclos K., Spencer R. M. C. (2013). Sleep spindles in midday naps enhance learning in preschool children. *Proceedings of the National Acadamy of Sciences of the United States of America*.

[22] Milner C. E., Fogel S. M., Cote K. A. (2006). Habitual napping moderates motor performance improvements following a short daytime nap. *Biological Psychology*.

[23] Lim J., Dinges D. F. (2010). A Meta-Analysis of the Impact of Short-Term Sleep Deprivation on Cognitive Variables. *Psychological Bulletin*.

[24] Almklov E. L., Drummond S. P. A., Orff H., Alhassoon O. M. (2015). The Effects of Sleep Deprivation on Brain Functioning in Older Adults. * Behavioral Sleep Medicine*.

[25] Chuah Y. M. L., Venkatraman V., Dinges D. F., Chee M. W. L. (2006). The neural basis of interindividual variability in inhibitory efficiency after sleep deprivation.. *The Journal of Neuroscience*.

[26] Drummond S. P. A., Paulus M. P., Tapert S. F. (2006). Effects of two nights sleep deprivation and two nights recovery sleep on response inhibition. *Journal of Sleep Research*.

[27] Renn R. P., Cote K. A. (2013). Performance monitoring following total sleep deprivation: Effects of task type and error rate. *International Journal of Psychophysiology*.

[28] Demos K. E., Hart C. N., Sweet L. H. (2016). Partial sleep deprivation impacts impulsive action but not impulsive decision-making. *Physiology & Behavior*.

[29] Hsieh S., Cheng I.-C., Tsai L.-L. (2007). Immediate error correction process following sleep deprivation. *Journal of Sleep Research*.

[30] Tsai L.-L., Young H.-Y., Hsieh S., Lee C.-S. (2005). Impairment of error monitoring following sleep deprivation. *SLEEP*.

[31] Balkin T. J., Bliese P. D., Belenky G. (2004). Comparative utility of instruments for monitoring sleepiness-related performance decrements in the operational environment. *Journal of Sleep Research*.

[32] Åkerstedt T., Gillberg M. (1990). Subjective and objective sleepiness in the active individual. *International Journal of Neuroscience*.

[33] Basner M., Rao H., Goel N., Dinges D. F. (2013). Sleep deprivation and neurobehavioral dynamics. *Current Opinion in Neurobiology*.

[34] de Bruin E. J., van Run C., Staaks J., Meijer A. M. (2017). Effects of sleep manipulation on cognitive functioning of adolescents: A systematic review. *Sleep Medicine Reviews*.

[35] Goel N., Rao H., Durmer J. S., Dinges D. F. (2009). Neurocognitive consequences of sleep deprivation. *Seminars in Neurology*.

[36] Jackson M. L., Gunzelmann G., Whitney P. (2013). Deconstructing and reconstructing cognitive performance in sleep deprivation. *Sleep Medicine Reviews*.

[37] Wickens C. D., Hutchins S. D., Laux L., Sebok A. (2015). The Impact of Sleep Disruption on Complex Cognitive Tasks. *Human Factors: The Journal of the Human Factors and Ergonomics Society*.

[38] Goldberg D., Bridges K., Duncan-Jones P., Grayson D. (1988). Detecting anxiety and depression in general medical settings. *British Medical Journal*.

[39] Roenneberg T., Wirz-Justice A., Merrow M. (2003). Life between clocks: daily temporal patterns of human chronotypes. *Journal of Biological Rhythms*.

[40] Beck A. T., Ward C. H., Mendelson M., Mock J., Erbaugh J. (1961). An inventory for measuring depression. *Archives of General Psychiatry*.

[41] Buysse D. J., Reynolds C. F., Monk T. H., Berman S. R., Kupfer D. J. (1989). The Pittsburgh Sleep Quality Index: a new instrument for psychiatric practice and research. *Psychiatry Research*.

[42] Eriksen B. A., Eriksen C. W. (1974). Effects of noise letters upon the identification of a target letter in a nonsearch task. *Perception & Psychophysics*.

[43] Backhaus J., Junghanns K. (2006). Daytime naps improve procedural motor memory. *Sleep Medicine*.

[44] Kaida K., Takeda Y., Tsuzuki K. (2012). The relationship between flow, sleepiness and cognitive performance: The effects of short afternoon nap and bright light exposure. *Industrial Health*.

[45] Zhang G., Sun F., Liao J. (2009). The effects of lunch time napping on habitual nappers mental work efficiency in the afternoon and early. *Health*.

[46] Hayashi M., Watanabe M., Hori T. (1999). The effects of a 20 min nap in the mid-afternoon on mood, performance and EEG activity. *Clinical Neurophysiology*.

[47] Takahashi M., Arito H. (2000). Maintenance of alertness and performance by a brief nap after lunch under prior sleep deficit. *SLEEP*.

[48] Tietzel A. J., Lack L. C. (2001). The short-term benefits of brief and long naps following nocturnal sleep restriction. *SLEEP*.

[49] Ru T., Chen Q., You J., Zhou G. (2017). Effects of a short midday nap on habitual nappers' alertness, mood and mental performance across cognitive domains. *Journal of Sleep Research*.

